# Activity Patterns and Functioning. A Contextual–Functional Approach to Pain Catastrophizing in Women with Fibromyalgia

**DOI:** 10.3390/ijerph18105394

**Published:** 2021-05-18

**Authors:** Cecilia Peñacoba, Maria Ángeles Pastor-Mira, Carlos Suso-Ribera, Patricia Catalá, Ainara Nardi-Rodríguez, Sofía López-Roig

**Affiliations:** 1 Department of Psychology, Rey Juan Carlos University, Avda. de Atenas s/n, 28922 Alcorcón, Madrid, Spain; cecilia.penacoba@urjc.es (C.P.); patricia.catala@urjc.es (P.C.); 2 Department of Behavioural Sciences and Health, Miguel Hernández University, Crtra Alicante-Valencia, km.8,7, 03550 San Juan, Alicante, Spain; mapastor@umh.es (M.Á.P.-M.); anardi@umh.es (A.N.-R.); 3 Department of Basic and Clinical Psychology and Psychobiology, Jaume I University, Vicent Sos Baynat, 15, 12006 Castellón de la Plana, Castellón, Spain; susor@uji.es

**Keywords:** fibromyalgia, psychological flexibility, activity patterns, functioning, pain catastrophizing, chronic pain

## Abstract

Background: The psychological flexibility model states that activity patterns are not deemed to be intrinsically functional or dysfunctional; it is considered that underlying factors, such as personal goals and contextual factors, are what will determine their effects on disability. Pain catastrophizing has frequently been associated with several important pain-related outcomes. Despite its recent conceptualization within affective–motivational approaches, its moderating role between activity patterns and dysfunction has not been analyzed. Methods: This study analyzes the moderating role of pain catastrophizing and its dimensions (Pain Catastrophizing Scale) between activity patterns (Activity Patterns Scale) and disease impact (Fibromyalgia Impact Questionnaire—Revised) in 491 women with fibromyalgia. Results: Activity avoidance (*p* < 0.001), excessive persistence (*p* < 0.001) and pacing (*p* < 0.01) patterns were positively associated with fibromyalgia impact. Helplessness shows a moderating role between pain avoidance (B = 0.100, t =2.30, *p* = 0.021, [0.01, 0.18]), excessive persistence (B = −0.09, t = −2.24, *p* = 0.02, [−0.18, −0.01]), pain persistence (B = −0.10, t = −2.04, *p* = 0.04, [−0.19, −0.004]) and functioning. Conclusion: Helplessness (within pain catastrophizing) is a relevant variable within psychological flexibility models applied to activity patterns. Specifically, pain avoidance is especially dysfunctional in patients with high helplessness. To improve excessive persistence and pain persistence, it is necessary to reduce helplessness before regulating activity patterns.

## 1. Introduction

A considerable amount of research supports cognitive-behavioral therapy (CBT) as the first line of psychosocial treatment for chronic pain [1]. Different systematic reviews and meta-analyses have revealed its effectiveness for reducing pain and pain interference, distress and disability [2,3], although the effect sizes are modest on average, especially in relation to pain indicators [4,5]. Therefore, and given that side effects of this therapeutic option are absent, it is of interest to study the processes involved in optimizing its results. The analysis of the results of CBT has highlighted different future research challenges [1] including aspects such as the identification, within multicomponent therapy, of the truly effective processes, the standardization of the measures throughout the different studies and the improvement of the adherence to the treatment. Within them, a matter of special interest is the fact that certain behavioral, cognitive and emotional variables can moderate the effects of CBT on patient outcomes, which raises the need to incorporate new explanatory models that in turn lead to the development of more effective and efficient therapies [6].

In this context, the model of psychological flexibility [7] provides the conceptual framework for the most recent psychological interventions in chronic pain (e.g., Acceptance and Commitment Therapy). CBT interventions for chronic pain have often focused on training patients in the use of adaptive coping strategies, including activity patterns to help reduce pain and its associated comorbidity, such as anxiety, depression or functional limitation [8]. However, to identify the ‘adaptive role’ of activity patters, previous research has indicated the need to conceptualize their effects from the perspective of psychological flexibility [9,10], exploring the variables of which explains the different effects that these patterns may have on outcomes, including disease impact [11]. The psychological flexibility model provides an alternative conceptual framework for interpreting the effect of activity patterns on daily functioning, allowing us to understand the absence of clear and consistent findings regarding the ‘linear’ effect of these patterns [9]. Far from linear and deterministic interpretations, the effect of activity patterns on chronic pain has to be understood from the perspective of complex and dynamic models, where the interference of disruptive pain on real-life physical activity, understood in terms of meaningful information, plays a fundamental role [12]. Psychological flexibility is considered to occur when we are able to persist or to change a behavior when there is a setting in which several psychological influences are competing, guided by goals and dependent on what the situation at hand affords [7,13]. Chronic pain not only interferes with performing everyday activities; it can also negatively affect a person’s perceived integrity, by creating self-discrepancies [14]. There is, in turn, an association of self-discrepancies with negative mood states, triggering self-regulatory behaviors aimed at reducing these discrepancies, projected in persistence or in avoidance patterns, depending on the type of self-discrepancies (ideal–other or feared–own self-discrepancies, respectively) [14]. In this context, activity patterns are not only the product of pain, but also of self-regulation of goals that an individual wants to achieve in the context of pain [15]. 

A greater knowledge of activity patterns is especially relevant in chronic pain patients, since their overall physical, social and emotional functioning are closely related to the level and pattern of daily activities performed [16,17]. From the fear of movement models, a person’s activity patterns in the face of pain (such as a stressful situation) provide an explanation for the development of chronic pain [17]. Specifically, the chronic pain literature has typically considered various approaches to activity, such as ‘activity avoidance’, ‘pacing’, and particular patterns of high-rate activity, called ‘persistence patterns’ [10,16,18]. ‘Activity avoidance’ has received great attention, as it has been suggested to be a maladaptive pattern linked to worse daily functioning [10,16,17,18,19]. However, the adaptive or maladaptive role of the rest of the activity patterns is not clearly defined [15,16,18,19], highlighting the need to analyze the effect of these patterns from the models of psychological flexibility previously commented.

‘Pacing’ patterns involve slowing down, breaking tasks into smaller pieces, or incorporating pauses in their performance in response to different personal goals, such as increasing activity levels, conserving energy for valued activities, or reducing pain [20,21]. ‘Persistence’ refers to continuing with an activity until completed, despite pain [18,22]. Within the persistence patterns, different approaches are included that contemplate both the motive that guides persistence (i.e., pain-contingent persistence, activity-contingent persistence) and the rigidity of the pattern itself (i.e., excessive persistence) [10,20,21]. It seems clear that the implications of these patterns cannot be considered outside of the models of psychological flexibility. Psychological flexibility includes processes such as reducing the struggle to control pain, increasing present-focused awareness, and improving participation in meaningful activities [7], among others; processes involved in activity patterns. Some of these processes have already been explicitly studied, such as the conceptualization of “committed action” [9], which refers to the importance of pursuing a life that is considered meaningful and consistent with one’s own values, which implies flexible persistence over time. Along the same line, Esteve et al. [20] incorporated the goal orientation of patterns as a differential characteristic that can define their adaptive or maladaptive character (i.e., three pacing patterns, each of which is associated with a single goal (conserving energy for valued activities, increasing activity levels, and reducing pain)). Finally, recent studies have pointed out the validity of the psychological flexibility model in the relationship between activity patterns and certain disease outcomes, highlighting the role of both contextual (i.e., pain intensity [23]) and personal variables associated with one’s values (i.e., perfectionism [24], goal preferences [25]).

Within the broader model of psychological flexibility, different processes and variables can be included, despite previous literature perhaps highlighting one above the others, namely pain acceptance [11]. Pain catastrophizing (PC) is defined as an exaggerated negative mental set that arises in response to present or anticipated pain, broken down into subcategories of rumination, magnification, and helplessness [26] constituting the psychosocial factor most frequently considered in predicting adjustment to chronic pain [27,28] through affective–motivational and cognitive–evaluative processes. Both processes can be studied from the models of psychological flexibility. From the affective–motivational perspective, people with chronic pain must manage multiple goals that may be incompatible (i.e., those related with resolving and avoiding pain and those associated with the maintenance, restoration or incorporation of other relevant goals, such as being active). In this context, pain is considered an obstacle to achieving other goals which are important to the person, and catastrophic thinking, especially the rumination component, a reflection of concern about that interference [29]. This affective–motivational perspective of PC connects directly with the model of psychological flexibility as it incorporates one’s personal goals and values as core variables [7,13]. From the cognitive–evaluative perspective, the psychological flexibility model highlights two types of variables that influence behavior: (1) environmental variables that include direct experience (i.e., pain), and (2) cognitive variables (verbal, language-based, or cognitive processes) [30]. From this perspective, PC, as a cognitive variable (in this case associated with a negative and exaggerated perception of the effects of pain), would have a place within the aforementioned models [31]. 

Different studies have analyzed PC along with the central elements of the psychological flexibility models [32], indicating that pain catastrophizing, mindfulness, and pain acceptance, although unique constructs, are related [33,34], therefore making it necessary to consider them together in explaining adaptation to chronic pain. Additionally, other studies have highlighted their relationship within more complex models, showing that pain acceptance can potentially mediate the association between pain catastrophizing and negative patient outcomes [35], emphasizing the need to analyze pain catastrophizing from a contextual-based perspective [36].

Pre–post changes in pain catastrophizing in different treatments for pain (i.e., CBT, active physical treatment, and CBT plus active physical therapy) have been found to be associated with improvements in functionality and symptoms [37,38]. However, little is known about its possible moderating role between activity patterns, pain and physical function from the perspective of psychological flexibility, taking into account that coping (i.e., activity patterns) is one of the main focuses of CBT interventions for chronic pain [8]. Minimal research has been conducted regarding the moderating role of certain variables that allow contextualizing the adaptive role of certain patterns. This type of research has been identified as one of the most relevant lines to optimize interventions in chronic pain [1]. 

In this context, the main objective of the present study has been to analyze the moderating role of pain catastrophizing between activity patterns and disease impact in women with fibromyalgia. This aim is posed while taking a more functional and contextual view of coping behaviors focused on the nature of interactions between behavior and situations where they occurs, on the influences being exerted on behavior, and the purposes being served (i.e., psychological flexibility model) [39]. To achieve this objective, the moderating role of each pain catastrophizing component (rumination, magnification and helplessness) has been analyzed. Different studies have pointed out the differential characteristics of the components of pain catastrophizing, revealing, additionally, its different relationships with disease outcomes in chronic pain [40]. Specifically, to our knowledge, no studies have been carried out that analyze the possible moderating role of catastrophism and its dimensions in the relationship between activity patterns and the impact of the disease. Despite the absence of studies in this regard, when proposing our hypotheses, we took into account two previous antecedents: (1) the results of the aforementioned studies that indicate the possible different mechanisms of each of the dimensions of pain catastrophizing in health outcomes. Previous literature in this regard is not excessively abundant, and the results found are not always consistent, so further research is necessary [41]. However, the results found indicate that rumination and helplessness seem to be linked to a wide diversity of health outcomes in chronic pain, while magnification seems to be more specifically related to worse mental health [41,42,43,44]. In this context, the Fibromyalgia impact measure (FIQR), used as an outcome in the present study, includes both indicators of interference of the disease and symptoms as pain, quality of sleep, and emotional symptoms, among others [45]. Therefore, a greater influence of rumination and helplessness are to be expected. (2) The previously mentioned affective–motivational perspective of pain catastrophism, which points to rumination as the most relevant dimension as a reflection of concern about goal interference caused by pain [29]. Consistent with these two previous antecedents, we anticipate that rumination and helplessness will be the dimensions that will moderate the relationship between activity patterns and disease impact, especially in pacing and persistence patterns (as they are more influenced by contextual and personal variables) [15,16,18,19]. 

## 2. Materials and Methods

### 2.1. Participants

The sample comprised 491 women with fibromyalgia diagnosed according to the American College of Rheumatology (ACR) criteria [46,47]. Following the minimum number criteria for modeling analysis [48], we stablished a minimum of 200 participants as sample size. This study is part of a larger project whose main objective is to identify the role of catastrophism, fear of pain and perception of self-efficacy in the preference and setting of goals related to exercise and walking behavior. Once participants had given informed consent to take part in the project, they were given a booklet of questionnaires that took 20 to 30 min to complete. The study followed the ethical principles for research with human participants and was approved by the University Committee on Ethics (blinded for review). Table 1 shows the socio-demographic and clinical characteristics of the sample. Mean age of the sample was 53.89 years old (SD = 9.25), which ranged from 19 to 78 years. Regarding the clinical variables, patients had experienced fibromyalgia for an average of 9.85 years (SD = 8.49; 1–46 years range). Most of the sample had been diagnosed in rheumatology units (70.6%, *n* = 331), only 10.2% (*n* = 48) had been diagnosed in primary care units, and a scarce 3% (*n* = 14) in traumatology units. The rest of the participants (16.2%, *n* = 76) had received their diagnoses in rehabilitation, neurology or pain and fibromyalgia units, among others. The average pain score was 7.05 (SD = 1.49; 1–10 range). More than half of the sample (55.8%, *n* = 387) regularly walk with the aim of doing physical exercise. Regarding the use of medication, the use of analgesics (including specific drugs for neuropathic pain such as gabapentin and pregabalin) was the most abundant (mean = 17.43 doses per week), followed by antidepressants (mainly serotonin noradrenaline reuptake inhibitors) (mean = 7.74 doses per week). The dose per week of sleeping pills (e.g., dipotassium chloracepate) was at 5.54, and that of muscle relaxants (e.g., diazepam or bromazepam) or other drugs (including treatments for hypertension, diabetes and irritable bowel, among others) was at 4.41 and at 11.19, respectively.

### 2.2. Measures

#### 2.2.1. Activity Patterns

The patterns (avoidance, pacing, and persistence) were assessed using the Activity Patterns Scale (APS) [20]. It has 24 items with a 5-point Likert-type response scale ranging from 0 (not at all) to 4 (always). The APS allows the assessment of eight types of activity patterns: pain avoidance (three items), activity avoidance (three items), task-contingent persistence (three items), excessive persistence (three items), pain-contingent persistence (three items) and three pacing patterns, each of which relates to a single goal (increasing activity levels, conserving energy for valued activities, and reducing pain) (three items each). For the purposes of this study and aiming for a parsimonious approach, the pacing pattern was considered in its entirety, according Kindermans et al. [10].

Specifically, avoidance can happen in two instances, one refers to the anticipation of changes in pain (i.e., pain avoidance) and the other to when the patient is in pain (activity avoidance). In relation to persistence, three differentiated patterns exist: the first would be task contingent persistence (i.e., when patients persist in finishing tasks or activities, despite pain), the second refers to excessive persistence (i.e., doing too much, not taking into consideration one’s physical limits), and the third is known as pain-contingent persistence (i.e., this occurs when the level of activity varies depending on and is determined by the pain in the moment). Finally, in relation to the pacing pattern, this allows patients to increase the time they need to complete tasks (breaking activities up into smaller parts, taking frequent short rests or slowing down) [20,49]. Previous research reveals a Cronbach’s alpha of 0.56 and 0.78 for pain avoidance, of 0.60 and 0.77 for activity avoidance, of 0.81 and 0.87 for task-contingent persistence, 0.69 and 0.80 for excessive persistence, 0.84 and 0.89 for pain-contingent persistence, and between 0.69 and 0.79 for pacing [20,21].

#### 2.2.2. Fibromyalgia Impact

To assess FM impact on functioning, the total score of the Spanish adaptation of the Fibromyalgia Impact Questionnaire-Revised (FIQ-R) was used [45]. The items in this scale are answered using an 11-point numerical scale, which ranges between 0 and 10, higher scores indicate higher impact of FM on functioning. Total FIQR scores range from 0 to 100. Spanish FIQR has shown high internal consistency (Cronbach’s α between 0.91 and 0.95) [45,50].

#### 2.2.3. Pain Catastrophizing

We used the Spanish adaptation of the Pain Catastrophizing Scale (PCS) [51]. This self-report is composed of 13 items that present a 5-point Likert response scale ranging from 0 (not at all) to 4 (always). Items measure three dimensions of pain catastrophizing: rumination (referring to repetitive thoughts about pain and the difficulty to inhibit them), magnification (threatening and exaggerated perception of pain and its consequences) and helplessness (perception of inability to cope with pain). The PCS is a widely used instrument with Cronbach’s alpha values ranging between 0.79 and 0.92 for total catastrophism, between 0.73 and 0.92 for rumination, between 0.61 and 0.74 for magnification, and between 0.73 and 0.90 for helplessness [51,52].

#### 2.2.4. Socio-Demographic and Clinical Data

To assess age, educational level, employment status, and marital status, an ad hoc questionnaire was used. In relation to the clinical variables, average pain levels and duration of fibromyalgia were recorded. The former was assessed using the mean score from four of the items: the minimum, maximum and overall pain intensity during the last 7 days, and pain intensity at the time of assessment. We used a numerical rating scale (0 = no pain and 10 = the worst pain you can imagine) for the four pain ratings [53]. 

### 2.3. Data Analysis

First, descriptive and bivariate Pearson correlation analyses were carried out. Given the multiple comparisons, Bonferroni correction was used to adjust for the risk of inflated error, considering *p*-values to be significant when below 0.01. Next, the moderation analyses were conducted using model 1 from the PROCESS Macro version 3.4 [54]. Pain catastrophizing and its dimensions were used as the moderator and activity patterns as independent variables (both variables were centered before the analyses). Centering consists of subtracting the mean from the predictors to rescale them. Centering has no effect on model fit (R2), significance tests, and standardized slope values. However, mean centering is recommended (but not mandatory) in moderation analyses to make the regression coefficients (intercepts) clearer. Specifically, by doing this the regression coefficients will reveal the effect when the remaining predictors have a value of ‘0’ [55]. This is useful in terms of interpretation, but of course creates ‘artificial’ scores by rescaling the predictors. Therefore, following past research [23], we will use centered variables when reporting regression coefficients. 

Twenty-four models were tested, complying with the six activity patterns (activity avoidance, pain avoidance, task-contingent persistence, excessive persistence, pain-contingent persistence and pacing) and the different dimensions of pain catastrophizing (total catastrophizing, rumination, magnification and helplessness). In all models, fibromyalgia impact was considered as an outcome. Statistical significance was set at an alpha level of 0.05. In the PROCESS Macro we followed the recommended values in conditional tables and graphical representations, which are the 16th, 50th, and 84th percentiles. When a moderation was found to be significant, these cut-offs were used to calculate conditional effects (i.e., effects of an independent variable on an outcome for different values of a moderator). Non-centered variables were used to facilitate the interpretation of results in the post hoc analyses. Uncentered variables (original scaling) reflect the ‘real’ scores with which readers will be familiar. 

## 3. Results

### 3.1. Means, Standard Deviations, and Pearson Correlations between Study Variables

Means, standard deviations, and Pearson correlations between study variables are presented in Table 2. Pain avoidance, activity avoidance, excessive persistence, and pain-contingent persistence maintained positive significant relationships with pain catastrophizing (all *p* < 0.001) and with its dimensions. Fibromyalgia impact showed positive significant relationships with pain catastrophizing and its dimensions (all *p* < 0.001). Fibromyalgia impact was found to have positive significant relationships with pacing (*p* = 0.004), pain avoidance (*p* = 0.001), activity avoidance (*p* < 0.001), excessive persistence (*p* < 0.001) and pain-contingent persistence (*p* = 0.005).

### 3.2. Regression Analyses Including Moderation 

The results of the regression analyses performed, including the analysis of moderation, can be found in Table 3 (for activity avoidance and pain avoidance), Table 4 (for task-contingent persistence and pain-contingent persistence) and Table 5 (for excessive persistence and pacing).

### 3.3. Activity Avoidance

The prediction of fibromyalgia impact from activity avoidance, pain catastrophizing, and their interaction (see Table 3) evidenced significant direct contributions of activity avoidance and pain catastrophizing and its dimensions. Beta values for pain catastrophizing dimensions ranged between 1.33 (helplessness) and 1.62 (magnification). Beta values for activity avoidance ranged between 1.70 (alongside helplessness) and 2.43 (alongside magnification). The models predicting fibromyalgia impact from activity avoidance, pain catastrophizing, and their interaction explained from 24% (alongside magnification) to 32% (alongside helplessness).

### 3.4. Pain Avoidance

The prediction of fibromyalgia impact from pain avoidance, pain catastrophizing, and their interaction (see Table 3) evidenced significant direct contributions (i.e., pain catastrophizing and its dimensions), but also a moderation of helplessness in the relationship between pain avoidance and fibromyalgia impact (Beta = 0.10, t = 2.30, *p* = 0.021, 95% IC = 0.01, 0.18). The models predicting fibromyalgia impact from pain avoidance, pain catastrophizing, and their interaction explained from 14% (alongside magnification) to 28% (alongside helplessness). 

**Figure 1:**
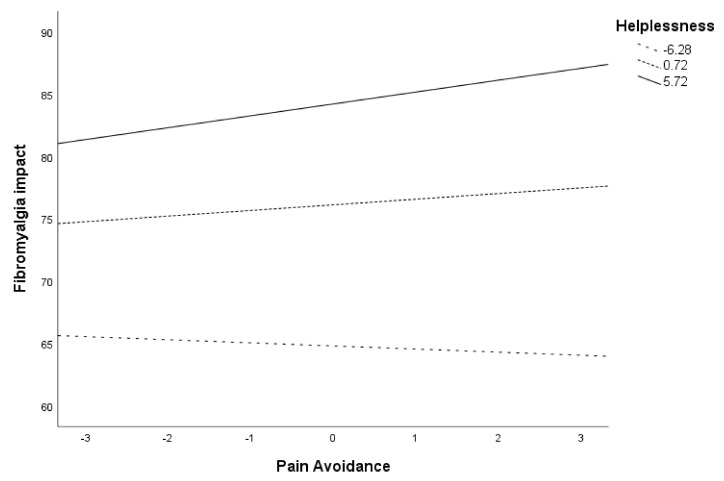
Conditional effects of pain avoidance on fibromyalgia impact at different values of helplessness.

As noted earlier, post hoc analyses were planned to analyze significant moderations more in-depth. These are presented in Table 6 and Figure 1 for the moderation of helplessness in the relationship between pain avoidance and fibromyalgia impact. As noted, both in Table 6 and Figure 1, the contribution of pain avoidance on fibromyalgia impact varied at different values of helplessness. Specifically, pain avoidance was only significantly associated with fibromyalgia impact when helplessness levels were high (Beta = 0.95, *p* = 0.006, 95% CI = 0.26, 1.64). When helplessness levels were moderate or low, the use of pain avoidance did not contribute to fibromyalgia impact (*p* = 0.101 and *p* = 0.552, respectively).

### 3.5. Task-Contingent Persistence

The prediction of fibromyalgia impact from task-contingent persistence, pain catastrophizing, and their interaction (see Table 4) evidenced significant direct contributions of pain catastrophizing and its dimensions. Beta values for pain catastrophizing dimensions ranged between 1.64 (helplessness) to 2.15 (magnification). The models predicting fibromyalgia impact from task-contingent persistence, pain catastrophizing, and their interaction explained between 14% (alongside magnification) and 27% (alongside helplessness).

### 3.6. Pain-Contingent Persistence

The prediction of fibromyalgia impact from pain-contingent persistence, pain catastrophizing, and their interaction (see Table 4) showed significant direct contributions (i.e., pain catastrophizing and its dimensions), but helplessness also moderated the relationship between pain-contingent persistence and fibromyalgia impact (Beta = −0.10, t = −2.04, *p* = 0.041, 95% IC = −0.19, −0.004). The models predicting fibromyalgia impact from pain-contingent persistence, pain catastrophizing, and their interaction explained between 14% (alongside magnification) and 28% (alongside helplessness).

**Figure 2:**
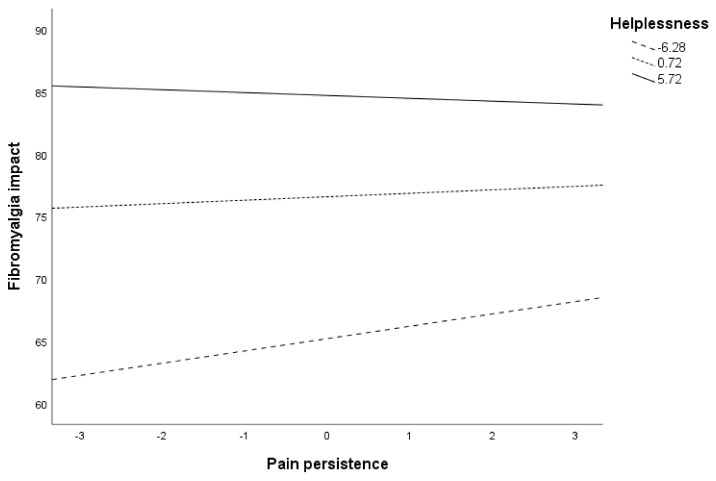
Conditional effects of pain persistence on fibromyalgia impact at different values of helplessness.

Post hoc analyses are presented in Table 7 and Figure 2 for the moderation of helplessness in the relationship between pain-contingent persistence and fibromyalgia impact. As noted, both in Table 7 and Figure 2, the contribution of pain-contingent persistence on fibromyalgia impact varied at different values of helplessness. Specifically, pain-contingent persistence was only significantly associated with fibromyalgia impact when helplessness levels were low (Beta = 0.99, *p* = 0.019, 95% CI = 0.16, 1.82). When helplessness levels were moderate or high, the use of pain-contingent persistence did not contribute to fibromyalgia impact (*p* = 0.374 and *p* = 0.595, respectively).

### 3.7. Excessive Persistence

The prediction of fibromyalgia impact from excessive persistence, pain catastrophizing, and their interaction (see Table 5) evidenced significant direct contributions (i.e., excessive persistence and pain catastrophizing and its dimensions), but also a moderation of helplessness in the relationship between excessive persistence and fibromyalgia impact (Beta = −0.10, t = −2.24, *p* = 0.025, 95% IC = −0.18, −0.01). The models predicting fibromyalgia impact from excessive persistence, pain catastrophizing, and their interaction explained between 17% (alongside magnification) and 30% (alongside helplessness).

**Figure 3:**
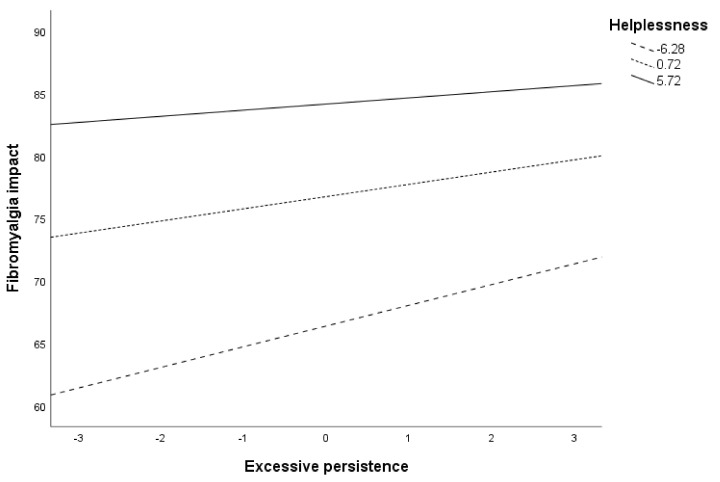
Conditional effects of excessive persistence on fibromyalgia impact at different values of helplessness.

Post hoc analyses are presented in Table 8 and Figure 3 for the moderation of helplessness in the relationship between excessive persistence and fibromyalgia impact. As noted, both in Table 8 and Figure 3, the contribution of excessive persistence on fibromyalgia impact varied at different values of helplessness. Specifically, excessive persistence was significantly associated with fibromyalgia impact when helplessness levels were low (Beta = 1.66, *p* < 0.001, 95% CI = 0.91, 2.40) or moderate (Beta = 0.98, *p* < 0.001, 95% CI = 0.42, 1.24). When helplessness levels were high, the use of excessive persistence did not contribute to fibromyalgia impact (*p* = 0.200).

### 3.8. Pacing

The prediction of fibromyalgia impact from pacing, pain catastrophizing, and their interaction (see Table 5) evidenced significant direct contributions of pacing and pain catastrophizing and its dimensions. Beta values for pain catastrophizing dimensions ranged between 1.63 (helplessness) and 2.11 (magnification). Beta values for pacing were similar in all cases, ranging between 0.22 and 0.26. The models predicting fibromyalgia impact from pacing, pain catastrophizing, and their interaction explained between 15% (alongside magnification) and 28% (alongside helplessness).

## 4. Discussion

The present study is the first to analyze, to our knowledge, the moderating role of pain catastrophizing and its dimensions in the relationship between activity patterns and disease impact. Given the relevance of pain catastrophizing as a predictor of pain [27,28] and the need to study the effect of activity patterns under models of psychological flexibility [9,10], it seems of interest to analyze the role of the former in the relationship between activity patterns and functionality in chronic pain. Specifically, our data indicate the contextual role of certain activity patterns (i.e., excessive persistence, pain avoidance and pain-related persistence). Their impact on the disease cannot be established a priori but it seems to depend on helplessness levels (as a dimension of pain catastrophizing). These results are consistent with the previous literature that indicates that the adaptive or maladaptive nature of the previous patterns, together with pacing, should be questioned [15,16,18,19].

In explaining this inconsistency, different variables have been analyzed in previous studies. The concept of goal, as a variable inherent to psychological flexibility [7,13], has been incorporated [25], showing that preference for pain avoidance goals has been consistently associated with pain and disability through different activity patterns, especially with regards to task-contingent persistence, excessive persistence and activity avoidance patterns. Other studies have studied the relationship between activity patterns and goal management strategies while also considering the roles of affectivity and optimism [15], showing that the latter can be associated with persistence, commitment to new goals, and flexible goal management, and is associated with persisting in finishing tasks despite pain, and can also show infrequent avoidance behavior when pain is anticipated. However, the contextual role, from the psychological flexibility model, of these variables in the relationship between activity patterns and functioning has not yet been sufficiently explored. Recent studies point out the role of pain and perfectionism as moderator variables in predicting the adaptive nature of certain activity patterns (i.e., pacing, excessive persistence, pain-contingent persistence, task-contingent persistence and pain avoidance) [23,24]. 

Regarding pain catastrophizing our results indicate the specific role of helplessness as a moderating variable. These results partially support the hypotheses raised. Thus, the role of helplessness is corroborated but not that of rumination. The role of rumination was fundamentally hypothesized due to its role within the characterization of pain catastrophizing from the affective–motivational perspective. This perspective suggests that people with chronic pain must manage multiple goals that may be incompatible, and therefore find themselves having to prioritize between the ones related to controlling pain and the ones associated with significant aspects of their life. Pain is considered an obstacle to achieve other important goals, and catastrophic thinking, mainly in its rumination component, a reflection of the concern for this interference. Thus, fear of pain would not only be the result of erroneous beliefs or highly threatening assessments regarding pain, but could also be the result of concern over the interference of pain in behaviors and efforts to achieve other significant goals. When the goal of alleviating or controlling pain is a requirement to achieve other vital goals, it ends up prevailing and gaining importance in the set of individual goals, promoting avoidance behaviors [29]. Furthermore, rumination constitutes the most analyzed factor within pain catastrophizing and some studies indicate that it has the greatest predictive power regarding the chronification of pain [42,43,56,57] and acute postsurgical pain [58]. 

Thus, the scarce existing literature on the specific mechanisms of action of rumination in the chronification of pain is focused on avoidance behavior. It could be hypothesized that its role is especially relevant in the impact of the disease when it is considered within activity avoidance patterns. Consistent with this approach, our results indicate significant and relevant direct effects of both rumination and activity avoidance patterns on the impact of the disease. It should not be forgotten, as it has been pointed out, that activity avoidance is considered in general terms as a maladaptive pattern [10,16,17,18,19], playing a fundamental role in fear of movement models of pain chronification [27]. Our results indicate likewise that this direct effect on the impact of the disease is also observed in the magnification component. Studies about magnification are less abundant, but its role has been associated with more affective motivational aspects such as short-term patient satisfaction with surgery [44], anxiety associated with painful endoscopy experiences [59], or its indirect effect on suicide risk through physical disability and depression [60], which could explain its role within the affective–motivational perspective of catastrophism linked to models of fear of movement.

The moderating role of helplessness, reflected in our initial hypothesis, is corroborated in our results. It is especially interesting that its role is significant in the activity patterns most subject to be explained from the psychological flexibility models (their adaptive or maladaptive nature cannot be determined a priori). This is, without a doubt, the most novel and relevant finding of the present study. Helplessness has previously been identified as a risk factor for both pain interference and pain severity in childhood trauma, 12 months after breast cancer surgery [61] and also in chronic pain patients with depression [62,63]. Different studies have been carried out specifically analyzing its role in chronic pain in adolescents, as a predictor variable of functioning improvement [64] and of persistent pain in idiopathic scoliosis [65]. Its role has been analyzed together with the fear-avoidance beliefs in postural stability in people with osteoarthritis [66]. Despite the absence, as has been pointed out, of specific studies on its moderating role in the relationship between activity patterns and disease impact, our results could be explained based on previous studies [64,66].

Helplessness refers to the perceived inability to cope with pain [41]. Our results indicate that at high levels of helplessness, both excessive persistence and pain-related persistence patterns are associated with greater fibromyalgia impact, regardless of the pattern’s frequency. If helplessness is low or medium, these patterns will only be maladaptive if they are used very frequently. In the case of the pain avoidance pattern, when helplessness is high, a significant positive relationship is observed between the pattern’s frequency and disease impact. Previous studies carried out with patients with fibromyalgia have pointed out the role of helplessness as the best predictor of the affective dimension of pain [67], and have highlighted its role in the impact of the disease, characterizing the moderate-to-high impact group [68]. Psychological flexibility facilitates the persistence or change of behavior based on personal goals and values, according to the interaction between mental processes and the environment [7]. It is likely that, in this context, helplessness constitutes the most representative element of catastrophizing, of not being able to have control over decision making.

Finally, with relative independence from the model of psychological flexibility, our results corroborate the maladaptive role of certain patterns, even when the effect of the catastrophizing, and its dimensions, has been accounted for. In addition to the aforementioned negative effect of activity avoidance, extensively documented in previous literature [10,16,17,18,19], our results point to the maladaptive role of excessive persistence and pacing. Regarding excessive persistence (i.e., overdoing), previous literature has shown its association with disability [10] and negative psychosocial and emotional outcomes [20,24,69,70]. Our results also point to the maladaptive role of pacing, although this data has not been sufficiently contrasted in the literature [18,19,21,69,71]. This maladaptive role is linked to its conceptualization as an avoidance pattern [16], which is oriented towards symptom reduction and is more pain-contingent rather than activity-contingent [10]. In relation to our results, it could be hypothesized that its direct maladaptive role could be explained by its consideration together with pain catastrophizing.

Certain shortcomings of this study should be mentioned. Due to its nature, the associations should be considered within its observational design. It is also important to take into account that the sample was composed of female fibromyalgia patients only, which does not guarantee the generalizability of the findings to other populations with chronic pain. Furthermore, the low internal consistency of the excessive persistence and of activity avoidance scales in both the current study and in past research should also be considered [20,21]. Future research should encourage a reformulation of the corresponding items in the scale. Finally, while we included important psychological mechanisms in pain research (i.e., pain catastrophizing), the concept of psychological flexibility is so broad that other variables could also be the object of study [11]. In fact, some patterns do not show direct effects on the impact of the disease and do not seem to depend on catastrophizing and its dimensions (i.e., task persistence), so the existence of other contextual variables could be hypothesized. We hope that the current study inspires similar research in the future about these and other psychological variables.

While acknowledging some limitations to our study, we should highlight that the present study’s findings have clinical implications that are of great importance in the field of personalized behavioral treatments for patients living with chronic pain [70], specifically with fibromyalgia. From the model of psychological flexibility, the present research highlights the relevance of helplessness within pain catastrophizing. The functionality of certain persistence patterns (pain-related persistence and excessive persistence) and pain avoidance is influenced by helplessness. In the case of persistence, reducing levels of helplessness before regulating the activity pattern seems a more appropriate intervention strategy. In the case of pain avoidance, both interventions on pattern regulation and on helplessness are equally indicated and are complementary. Furthermore, given the multidimensional nature of pain catastrophizing [41], it is especially interesting to direct actions towards the specific component involved. As has been pointed out, although pain catastrophizing has been considered as one of the most relevant psychological predictors of chronicity and disability related to pain, there are important problems still remaining in relation to its conceptualization [72]. Clarifying the construct and analyzing the elements involved in each case is essential for the development of more personalized, and consequently, more effective interventions [72]. This multidimensionality could explain the fact that most interventions aimed at modifying pain catastrophizing produce only modest benefits, unless targeted to people showing high levels of this variable [31]. The analysis of the literature has indicated different strategies to decrease pain catastrophizing including stop thinking, rational-emotive therapy, emotional regulation, behavioral inhibition and behavioral activation (BIS/BAS) systems, acceptance and commitment therapy, and interoceptive sensitivity [72]. Further investigation regarding patient profiles will result in greater efficacy and efficiency of treatments.

## 5. Conclusions

This study reveals the moderating role of helplessness (as a dimension of pain catastrophizing) between some activity patterns (i.e., excessive persistence, pain avoidance and pain-related persistence) and disease impact. These results allow for further advances in changing the way we study activity patterns, from more traditional models to models of psychological flexibility [7,9,13], taking a more contextual and functional perspective [16]. Our results support the idea that rather than increasing/diminishing the frequency of certain behaviors (i.e., activity patterns), it would be desirable for interventions to focus on the interactions between the behavior and the situations in which it occurs, taking into account the influences exerted (i.e., helplessness) and the goals pursued [11]. As Ehde et al. [1] point out, this change in the model implies a change from the question “Does it work?” to the question “Why, for whom, and under what circumstances does it work?” In short, our results show that further research on moderators is essential to understand the results of certain behaviors (such as activity patterns) on health/disease outcomes, and consequently, to contribute to the design and implementation of more effective treatments [1].

## Figures and Tables

**Table 1:** Sociodemographic and clinical data of the sample (*n* = 491).

	*n* (%)
Education level	
No studies	61 (12.4)
Primary	258 (52.5)
Secondary	135 (27.5)
University	37 (7.6)
Marital Status	
Married	368 (74.9)
Single	38 (7.7)
Widowed/separated	85 (17.3)
Employment status	
Currently employed	111 (22.6)
Unemployed	77 (15.7)
Sick leave	77 (15.7)
Retired	48 (9.8)
Retired due to disability	65 (13.2)
Housewife	113 (22.9)
Medication use (dose per week)	Mean (SD)
Analgesics	17.43 (13.58)
Sleeping pills	5.54 (6.66)
Antidepressants	7.74 (8.71)
Muscle relaxants	4.41 (7.23)
Other drugs	11.19 (11.58)

**Table 2:** Cronbach’s alpha, means, standard deviations, and Pearson correlations between study variables.

	Cronbach’s Alpha	Mean (SD)	2	3	4	5	6	7	8	9	10	11
1. Pain catastrophizing	0.94	30.20 (12.17)	0.92 **	0.87 **	0.95 **	0.19 **	0.39 **	−0.02	0.34 **	0.16 **	0.05	0.49 **
2. Rumination	0.89	9.68 (4.22)		0.73 **	0.81 **	0.19 **	0.33 **	−0.01	0.28 **	0.13 **	0.02	0.43 **
3. Magnification	0.80	6.23 (3.15)			0.75 **	0.18 **	0.28 **	−0.03	0.32 **	0.15 **	0.04	0.52 **
4. Helplessness	0.90	14.28 (5.81)				0.16 **	0.42 **	−0.03	0.32 **	0.15 **	0.05	0.52 **
5. Pain avoidance	0.72	6.61 (2.58)					0.35 **	−0.49 **	−0.16 **	−0.07	0.57 **	0.15 **
6. Activity avoidance	0.63	7.41 (2.53)						−0.26 **	0.09	0.08	0.31 **	0.41 **
7. Task-contingent persistence	0.81	6.72 (2.75)							0.46 **	0.30 **	−0.39 **	−0.05
8. Excessive persistence	0.62	7.07 (2.63)								0.43 **	−0.16 **	0.31 **
9. Pain-contingent persistence	0.79	9.25 (2.34)									0.05	0.13 **
10. Pacing	0.92	6.82 (2.72)										0.13 **
11. Fibromyalgia impact	0.94	75.21 (18.42)										

** *p* < 0.01.

**Table 3:** Prediction of fibromyalgia impact from activity avoidance, pain avoidance, pain catastrophizing (total score and dimensions), and their interaction.

	Beta	t	95% CI			Beta	t	95% CI	
VD: FIQR				R^2^ = 0.30 F = 70.42 ***	VD: FIQR				R^2^ = 0.25 F = 54.51 ***
Activity avoidance	1.89	6.33 ***	1.30, 2.48		Pain avoidance	0.32	1.13	−0.23, 0.89	
Pain catastrophizing	0.59	9.56 ***	0.47, 0.71		Pain catastrophizing	0.73	12.1 ***	0.61, 0.85	
Interaction	−0.01	−0.52	−0.05, 0.03		Interaction	0.04	1.77	<0.01, 0.08	
VD: FIQR				R^2^ = 0.27 F = 59.41 ***	VD: FIQR				R^2^ = 0.19 F = 38.46 ***
Activity avoidance	2.21	7.41 ***	1.62, 2.80		Pain avoidance	0.44	1.48	−0.14, 1.03	
Rumination	1.43	7.99 ***	1.08, 1.78		Rumination	10.83	10.1 ***	1.47, 2.19	
Interaction	−0.01	−0.23	−0.13, 0.11		Interaction	0.08	1.21	−0.05, 0.19	
VD: FIQR				R^2^ = 0.24 F = 52.10 ***	VD: FIQR				R^2^ = 0.14 F = 27.57 ***
Activity avoidance	2.43	8.14 ***	1.84, 3.02		Pain avoidance	0.54	1.79	−0.05, 1.15	
Magnification	1.62	6.78 ***	1.15, 2.09		Magnification	20.06	8.30 ***	1.57, 2.55	
Interaction	−0.05	−0.65	−0.22, 0.11		Interaction	0.09	1.01	−0.08, 0.26	
VD: FIQR				R^2^ = 0.32 F = 75.50 ***	VD: FIQR				R^2^ = 0.28 F = 63.88 ***
Activity avoidance	1.70	5.66 ***	1.11, 2.29		Pain avoidance	0.38	1.36	−0.16, 0.93	
Helplessness	1.33	10.2 ***	1.07, 1.59		Helplessness	10.61	13.1 ***	1.37, 1.85	
Interaction	−0.03	−0.62	−0.11, 0.06		Interaction	0.10	2.30 *	0.01, 0.18	

VD: Dependent variable; FIQR: Fibromyalgia Impact; * *p* <.05; *** *p* < 0.001.

**Table 4:** Prediction of fibromyalgia impact from task-contingent persistence and pain-contingent persistence, pain catastrophizing (total score and dimensions), and their interaction.

	Beta	t	95% CI			Beta	t	95%CI	
VD: FIQR				R2 = 0.24 F = 52.82 ***	VD: FIQR				R2 = 0.25 F = 54.21 ***
Task persistence	−0.27	−1.03	−0.79,0.24		Pain persistence	0.32	1.12	−0.26,0.97	
Pain catastrophizing	0.74	12.4 ***	0.63,0.86		Pain catastrophizing	0.73	12.2 ***	0.61,0.85	
Interaction	−0.01	−0.30	−0.04,0.03		Interaction	−0.04	−1.68	−0.09,0.01	
VD: FIQR				R2 = 0.19 F = 37.35 ***	VD: FIQR				R2 = 0.19 F = 38.66 ***
Task persistence	−0.33	−1.19	−0.87,0.21		Pain persistence	0.57	1.76	−0.06,1.21	
Rumination	1.87	10.5 ***	1.52,2.22		Rumination	1.83	10.2 ***	1.47,2.18	
Interaction	<0.00	−.05	−0.12,0.12		Interaction	−0.09	−1.25	−0.24,0.05	
VD: FIQR				R2 = 0.14 F = 26.16 ***	VD: FIQR				R2 = 0.14 F = 27.40 ***
Task persistence	−0.28	−1.01	−0.84,0.27		Pain persistence	0.47	1.39	−0.19,1.13	
Magnification	2.15	8.76 ***	1.67,2.63		Magnification	2.12	8.51 ***	1.63,2.61	
Interaction	<0.00	.01	−0.16,0.16		Interaction	−0.14	−1.32	−0.34,0.07	
VD: FIQR				R2 = 0.27 F = 61.29 ***	VD: FIQR				R2 = 0.28 F = 63.88 ***
Task persistence	−0.24	−0.93	−0.74,0.27		Pain persistence	0.34	1.14	−0.25,0.95	
Helplessness	1.64	13.4 ***	1.40,1.88		Helplessness	1.62	13.2 ***	1.38,1.87	
Interaction	−0.05	−1.16	−0.13,0.03		Interaction	−0.10	−2.04 *	−0.19, <0.01	

VD: Dependent variable; FIQR: Fibromyalgia Impact; * *p* < 0.05; *** *p* < 0.001.

**Table 5:** Prediction of fibromyalgia impact from excessive persistence and pacing, pain catastrophizing (total score and dimensions), and their interaction.

	Beta	t	95% CI			Beta	t	95% CI	
VD: FIQR				R^2^ = 0.27 F = 59.22 ***	*VD: FIQR*				R^2^ = 0.25 F = 55.58 ***
Exces. persistence	1.08	3.75 ***	0.51, 1.65		Pacing	0.23	2.52*	0.05, 0.40	
Pain catastrophizing	0.66	10.6 ***	0.54, 0.79		Pain catastrophizing	0.74	12.4 ***	0.62, 0.85	
Interaction	−0.02	−1.01	−0.06, 0.02		Interaction	<0.01	0.50	<0.01, 0.02	
*VD: FIQR*				R^2^ = 0.22 F = 46.26 ***	*VD: FIQR*				R^2^ = 0.20 F = 40.49 ***
Exces. persistence	1.40	4.81 ***	0.83, 1.97		Pacing	0.26	2.72 **	0.07, 0.44	
Rumination	1.62	8.93 ***	1.27, 1.98		Rumination	1.86	10.5 ***	1.51, 2.20	
Interaction	<0.01	−0.06	−0.12, 0.12		Interaction	0.01	0.62	−0.03, 0.05	
*VD: FIQR*				R^2^ = 0.17 F = 34.55 ***	*VD: FIQR*				R^2^ = 0.15 F = 28.32 ***
Exces. persistence	1.45	4.74 ***	0.85, 2.06		Pacing	0.22	2.30 *	0.03, 0.41	
Magnification	1.76	6.94 ***	1.26, 2.26		Magnification	2.11	8.66 ***	1.63, 2.59	
Interaction	0.02	0.19	−0.15, 0.18		Interaction	0.02	0.75	−0.03, 0.07	
*VD: FIQR*				R^2^ = 0.30 F = 69.28 ***	*VD: FIQR*				R^2^ = 0.28 F = 63.66 ***
Exces. persistence	1.05	3.73 ***	0.49, 1.59		Pacing	0.23	2.60 **	0.05, 0.40	
Helplessness	1.48	11.6 ***	1.23, 1.72		Helplessness	1.63	13,3 ***	1.39, 1.87	
Interaction	−0.10	−2.24 *	−0.18, −0.01		Interaction	<0.01	0.44	−0.02, 0.03	

VD: Dependent variable; FIQR: Fibromyalgia Impact; * *p* < 0.05; ** *p* < 0.01; *** *p* < 0.001.

**Table 6:** Conditional effects of pain avoidance on fibromyalgia impact at values of helplessness.

Helplessness	Beta (Pain Avoidance)	t	p	95% CI
−6.28	−0.25	−0.60	0.552	−1.06, 0.56
0.72	0.45	1.63	0.101	−0.09, 0.99
5.71	0.95	2.72	0.006	0.26, 1.64

**Table 7:** Conditional effects of pain persistence on fibromyalgia impact at different values of helplessness.

Helplessness	Beta (Pain Persistence)	t	p	95% CI
−6.28	0.99	2.34	0.019	0.16, 1.82
0.72	0.28	0.89	0.374	−0.33, 0.89
5.71	−0.23	−0.53	0.595	−1.08, 0.62

**Table 8:** Conditional effects of excessive persistence on fibromyalgia impact at different values of helplessness.

Helplessness	Beta (Excessive Persistence)	t	*p*	95% CI
−6.28	1.66	4.35	<0.001	0.91, 2.40
0.72	0.98	3.44	<0.001	0.42, 1.53
5.72	0.49	1.28	0.200	−0.26, 1.24

## Data Availability

The data presented in this study are available on request from the corresponding author. The data are not publicly available due to privacy restrictions.
